# Apolipoprotein Mimetic Peptides: Potential New Therapies for Cardiovascular Diseases

**DOI:** 10.3390/cells10030597

**Published:** 2021-03-08

**Authors:** Anna Wolska, Mart Reimund, Denis O. Sviridov, Marcelo J. Amar, Alan T. Remaley

**Affiliations:** Lipoprotein Metabolism Laboratory, Translational Vascular Medicine Branch, National Heart, Lung, and Blood Institute, National Institutes of Health, Bethesda, MD 20892, USA; mart.reimund@nih.gov (M.R.); sviridovd@nhlbi.nih.gov (D.O.S.); mamar@nhlbi.nih.gov (M.J.A.); aremaley1@nhlbi.nih.gov (A.T.R.)

**Keywords:** apolipoprotein, atherosclerosis, cardiovascular diseases, cholesterol, clinical trials, HDL, lipoproteins, mimetic, peptides, therapy

## Abstract

Since the seminal breakthrough of treating diabetic patients with insulin in the 1920s, there has been great interest in developing other proteins and their peptide mimetics as therapies for a wide variety of other medical disorders. Currently, there are at least 60 different peptides that have been approved for human use and over 150 peptides that are in various stages of clinical development. Peptides mimetic of the major proteins on lipoproteins, namely apolipoproteins, have also been developed first as tools for understanding apolipoprotein structure and more recently as potential therapeutics. In this review, we discuss the biochemistry, peptide mimetics design and clinical trials for peptides based on apoA-I, apoE and apoC-II. We primarily focus on applications of peptide mimetics related to cardiovascular diseases. We conclude with a discussion on the limitations of peptides as therapeutic agents and the challenges that need to be overcome before apolipoprotein mimetic peptides can be developed into new drugs.

## 1. Introduction

“Apo” the root for the word apolipoprotein is from ancient Greek and means “away from”. Hence, the word apolipoprotein (apo) is a fitting term for proteins that are purified away from lipoproteins, the micellar-like particles that transport water-insoluble lipids in the plasma compartment of the blood [1]. Most apolipoproteins have a structural role in maintaining the integrity of lipoproteins, but in some instances, they also affect lipoprotein metabolism by serving as ligands for lipoprotein receptors or by modulating the activity of the various plasma lipid modifying enzymes [2].

Apolipoproteins exist in two general classes: non-exchangeable and exchangeable [3]. The only non-exchangeable apolipoprotein is apolipoprotein B (apoB), which permanently resides on triglyceride-rich lipoproteins (TRL), which are chylomicrons (CM), very low-density lipoproteins (VLDL) and low-density lipoproteins (LDL) as one copy per particle. It is its β-sheet secondary structure that allows apoB to tightly bind to lipids, which accounts for its non-exchangeability. The remaining apolipoproteins are considered as exchangeable, because they are in dynamic equilibrium between being bound to lipids on lipoproteins or being free in a lipid-free or lipid-poor state [4]. Exchangeable apolipoproteins can equilibrate between all the various types of lipoproteins, such as those containing apoB, as well as high-density lipoproteins (HDL). Due to the higher radius of curvature of HDL, which results in phospholipid packing defects, the exchangeable lipoproteins are, in general, more enriched on HDL than the larger apoB-containing lipoprotein particles [5]. The main secondary structure on the exchangeable apolipoproteins is the amphipathic helix, which accounts for the ability of these proteins to bind to lipids [6].

In this review, we will discuss how short synthetic peptide mimetics of the exchangeable apolipoproteins have been developed first as tools for better understanding lipoprotein metabolism and then later as possible therapeutic agents for cardiovascular (CV) diseases. Although there are more than a dozen different apolipoproteins, we will focus on three of the most important and well-studied peptide mimetics, namely those based on apolipoprotein A-I (apoA-I), apolipoprotein E (apoE), and apolipoprotein C-II (apoC-II). Due to the broad range of this topic, we will mostly emphasize the principles behind the design of apolipoprotein mimetic peptides by discussing select examples. We will also summarize the latest efforts related to the testing of apolipoproteins and their peptide mimetics in clinical trials for CV diseases.

## 2. ApoA-I

### 2.1. ApoA-I Biochemistry

ApoA-I is the main protein on HDL (Table 1) and comprises about 70% of the total protein mass of HDL [7]. It is synthesized by the liver and intestine and it is one of the most abundant plasma protein, with a concentration range of 120–140 mg/dL (43–50 μM). ApoA-I is 28.3 kDa in molecular weight and contains ten α-helices [8]. Most of the helices are class A amphipathic α-helices, which are characterized by a large hydrophobic face and a polar face with negatively charged amino acids residues in the center and positively charged amino acids at the boundary between the hydrophobic and polar face [6]. This is the structural feature that enables exchangeable apolipoproteins to bind to lipids. The hydrophobic face is buried in the acyl chains of phospholipids on the surface of the lipoprotein particle and the positively charged amino acids like lysine and arginine form ionic interactions with the negatively charged head groups on phospholipids. The negatively charged amino acids like glutamic or aspartic acid or polar amino acids like serine interact with the aqueous environment. Depending on the size of HDL, there are between 2–5 copies of apoA-I per HDL particle.

**Figure 1:**
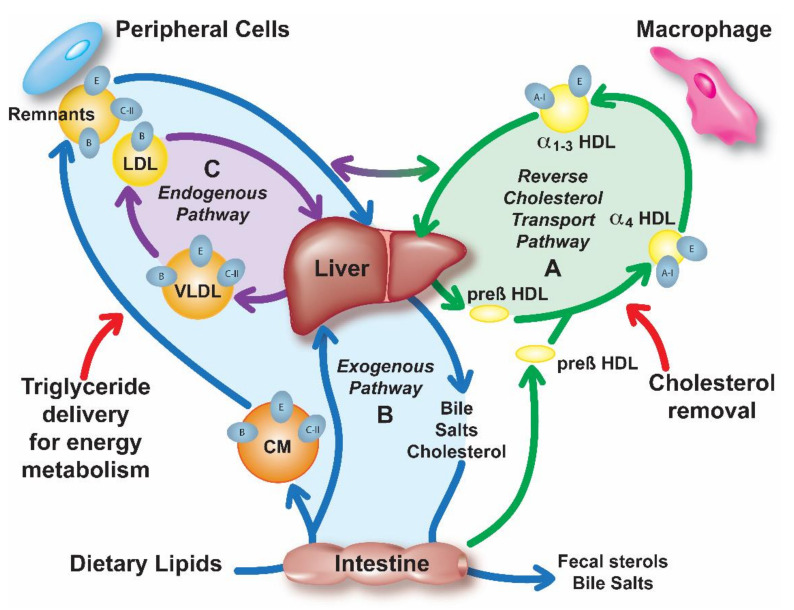
Overview of lipoprotein metabolism. Lipoprotein metabolism consists of three pathways: (**A**) Reverse cholesterol transport (RCT) (green); High-density lipoproteins (HDL) remove excess cellular cholesterol from peripheral tissues and macrophages and deliver it to the liver for excretion into the bile or for reutilization. (**B**) Exogenous pathway (blue); Dietary lipids are absorbed in the intestine and secreted into circulation in a form of chylomicron (CM) particles, which contain apoB-48, apoE and apoC-II. Triglycerides (TG) of CM are hydrolyzed by lipoprotein lipase (LPL), which requires apoC-II, forming remnant particles that are removed by the liver by apoE-mediated binding to low-density lipoproteins (LDL)-receptor (LDLR), LDLR-related protein (LRP) or heparan sulfate proteoglycan (HSPG). Free fatty acids from TG lipolysis are taken up by the peripheral tissues for energy production or storage. (**C**) Endogenous pathway (purple); Very low-density lipoproteins (VLDL) containing TG in their core and also apoB-100, apoE and apoC-II are secreted from the liver into circulation where they undergo LPL lipolysis forming remnants and LDL particles, which are eventually returned to the liver through interaction with various receptors or HSPG.

The most investigated putative atheroprotective function of apoA-I is its ability for the reverse cholesterol transport (RCT) (Figure 1A), the pathway by which excess cellular cholesterol is removed from peripheral tissues and delivered to the liver for excretion or reutilization [11]. ApoA-I is also known to have anti-inflammatory [12] and anti-oxidative [13] properties that could possibly be more relevant than RCT to its biological effects on atherosclerosis [14]. A discoidal form of HDL with two copies of apoA-I called pre-β HDL and possibly other lipid-poor forms of apoA-I, interact with cell membranes of macrophages and other cells and acquire cholesterol and phospholipids from ATP binding cassette 1 transporter (ABCA1)-dependent lipid microdomains by a detergent-like extraction process [15]. Initially, the cholesterol extracted by apoA-I resides in the outer phospholipid monolayer of HDL, but it can be esterified by lecithin:cholesterol acyl transferase (LCAT), which is activated by apoA-I. Due to its increased hydrophobicity, cholesteryl ester enters into the central hydrophobic core of HDL, converting it to larger more spherical α-migrating species of HDL (αHDL) (Figure 1A). Cholesteryl esters on HDL can then be directly delivered to the liver via scavenger receptor class B type 1 (SR-B1) or transferred to LDL by cholesteryl ester transfer protein (CETP) and later removed from the circulation via the LDL-receptor (LDLR). The mechanism for the other potential beneficial properties of apoA-I, such as its anti-inflammatory and anti-oxidative effects, are not as well understood, but in many instances it may still be related to the ability of HDL to alter the cholesterol content of cells, which has many pleiotropic effects on cell function [16].

### 2.2. ApoA-I Mimetic Peptide Design

**Figure 2:**
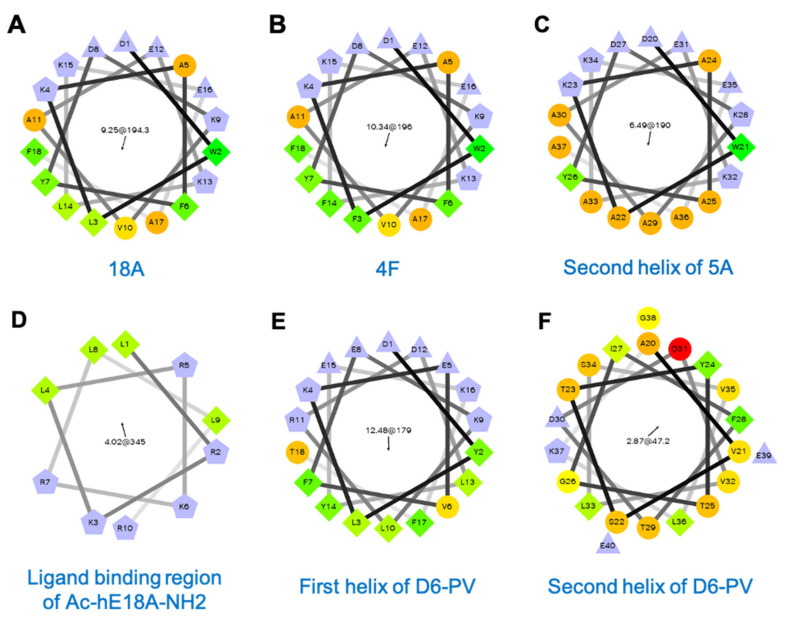
Helical wheel plots of apolipoprotein mimetic peptides: (**A**) 18A, (**B**) 4F, (**C**) Second helix of 5A, (**D**) ligand-binding region of Ac-hE18A-NH2, (**E**) First helix of D6PV, (**F**) Second helix of D6PV. First number in the center of the helical wheel plot indicates hydrophobic moment and the second number with a black arrow shows an angle of the hydrophobic moment. Purple: charged residues, Green: hydrophobic residues, Orange: polar or partially hydrophobic amino acids.

ApoA-I mimetic peptides have largely been designed based on their ability to efflux cholesterol from cells (Table 2). As this process has not been shown to depend upon a specific protein-protein interaction [17], most apoA-I mimetic peptides are simply just amphipathic helices and, in fact, many have no primary amino acid homology to apoA-I. The first of these peptides were designed in 1980s as structural probes for understanding lipoprotein assembly. The 18A peptide (Figure 2A) is an early example of such peptides, and its 18 amino acids are arranged to form a class A amphipathic helix. Several salt bridges between positively and negatively charged residues in the polar face that are 3–4 residues apart also help stabilize helix formation [18]. The ability of 18A to bind to lipids and to act like a detergent in interacting with lipids was shown by the solubilization of small unilamellar vesicles made of 1,2-dimyristoyl-sn-glycero-3-phosphocholine (DMPC) [19]. Blocking N- and C-termina with acetyl and NH_2_ groups, respectively, increased its lipophilicity and helicity, by promoting the hydrogen bond formation of the peptide backbone [20].

The 4F peptide (Figure 2B) is closely related to 18A, but it has two helices linked by proline and has more (total of four) phenylalanine residues in its hydrophobic face and hence its name [27]. When 4F is administered intraperitoneally (IP) or intravenously (IV), it has potent anti-atherogenic effects in numerous animal models [28]. It also has anti-inflammatory, anti-oxidant and other beneficial effects in a wide variety of disease models, such as for influenza A pneumonia [29], sickle cell-induced vascular dysfunction [30], scleroderma [31], type I and type II diabetes [32,33], hepatic fibrosis [34], Alzheimer’s disease [35], arthritis [36], hyperlipidemia-induced renal inflammation [37], and lipopolysaccharide (LPS)-induced acute lung injury [38]. Interestingly, D-4F, the 4F analog made with D-isomers of amino acids, displayed similar biological properties [39], demonstrating again that the most important attribute of apoA-I mimetics is their helicity and amphipathicity. Unlike its L-stereoisomer, D-4F has some limited oral bioavailability, because it is resistant to proteolysis in the intestine and was shown in animal models to reduce atherosclerosis when given orally [40]. Another closely related bi-helical peptide is 5A. It has five alanine residues in its second helix (Figure 2C), which was shown to make it more specific for removing cholesterol by the ABCA1 transporter and for being less cytotoxic [41]. Finally, another early apoA-I mimetic peptide, which was designed to not only form an amphipathic helix but to specifically activate LCAT, is ETC-642 (Table 2) [42]. It has also been shown to be atheroprotective in animal models [43,44].

More recently, many other apoA-I mimetic peptides have been described [45,46]. For example, an amphipathic peptide called FAMP with a single 24 amino acids helix was developed in Fukuoka University [47]. It was recently modified to i-FAMP-D1 by the addition of D-alanine to C-terminus to increase its plasma half-life [48]. Both peptides showed good cholesterol efflux ability based on in vitro studies and reduced atherosclerotic lesions in ApoE-KO mice. Some of the newer apoA-I mimetics utilize hydrocarbon staples [49] or α-methylated amino acids [50] to stabilize their amphipathic helices. These structural changes also make these peptides resistant to proteolysis [49,50]. Other newer apoA-I mimetics use a different backbone, such as polyproline [51], which forms a very stable left-handed helix [52]. To the proline ring, hydrophobic and polar groups were attached so that it forms an amphipathic helix and short polypro peptides containing as few as nine residues were shown to be effective for removing cholesterol from cells by the ABCA1 transporter [51]. Short self-assembling cyclic d,l-α-peptides have also been described to promote cellular cholesterol efflux from cells and to increase plasma levels of pre-β HDL in animal models [53].

### 2.3. ApoA-I Mimetic Peptide Clinical Trials

Prior to the testing of apoA-I mimetic peptides (Table 2), several different formulations of either recombinant [54,55,56] or purified full-length apoA-I [57] have been tested in clinical trials after complexation with phospholipids to make synthetic HDL (Table 3) [58]. In small early stage trials that were mostly based on CV imaging endpoints, such as intravascular ultrasound (IVUS), a small number of intravenous infusions over a period of only a few weeks appeared to have a favorable effect in rapidly reducing atherosclerotic plaque volume [54,59]. Subsequently, several much larger studies have failed to show benefit for this type of therapy [55,60,61]. Currently, there is only one apoA-I type therapy, CSL-112, in active clinical development, which is being tested in a phase 3 trial (AEGIS-II) (Table 3) [57]. In vitro and in healthy subjects, CSL-112 results in a dose-dependent increase in apoA-I, pre-β HDL levels and overall increase in RCT parameters [60,61]. In the AEGIS-II study, 17,400 patients with acute coronary syndrome are randomized to receive IV either CSL-112 (Table 3) or a placebo and the time to first occurrence of any component of major adverse CV events is the primary endpoint. It is expected to be completed by mid-2023.

In regard to apoA-I mimetic peptides, the first tested in a phase 1 clinical trial was the 4F peptide made with D-amino acids (D-4F) (Table 2). D-4F was given orally but the bioavailability was less than 1% [22]. It did not show any effect on HDL-cholesterol (HDL-C) levels [22]. Nevertheless, it improved the anti-inflammatory activity of HDL as measured in a monocyte chemotaxis assay. In another phase 1 trial, the intravenous infusion of the 4F peptide made with L-amino acids (L-4F) (Table 2) resulted in much higher peptide plasma levels than oral D-4F (Table 2) but showed no effect in improving the anti-inflammatory function of HDL or in modulating HDL-C levels [23]. It has been hypothesized that the benefit from apoA-I mimetic peptides may relate to their ability to sequester and prevent the absorption of oxidized dietary lipids [63]. The ETC-642 peptide (Table 2), which is the amphipathic peptide that was designed to activate LCAT [44] has also been tested in a phase 1 clinical trial but the results have not yet been reported [64].

Currently, the only apoA-I mimetic in active clinical development is the 5A peptide complexed with sphingomyelin (Fx-5A). Similar to 4F, it has been shown to be effective in reducing atherosclerosis in animal models [26] and to have anti-inflammatory effects in other animal models [65,66,67]. It has undergone pre-investigational new drug toxicology studies [68] and is now being tested in a phase 1 clinical trial [69].

## 3. ApoE

### 3.1. ApoE Biochemistry

ApoE is expressed in numerous tissues and cell types, including macrophages in the artery wall, but the predominant site of synthesis is the liver (Table 1) [70,71]. Mature human apoE contains 299 amino acids and has a molecular weight of 34 kDa. It is *O*-glycosylated at threonine 194. Plasma apoE levels are approximately 4–7 mg/dL (1–2 µM) and are mainly bound to CM remnants, VLDL and HDL [72]. It facilitates the hepatic clearance of TRL remnants (Figure 1B,C) by acting as a ligand for the LDLR [71,73]. It also interacts with other receptors, such as the LDLR-related protein (LRP) [74], and also heparan sulfate proteoglycans (HSPG) on cells’ surfaces [75,76,77]. As it contains several amphipathic helices, apoE can also mediate cholesterol efflux (Figure 1A) [70,78,79] and like apoA-I it has anti-inflammatory and anti-oxidative properties [80,81].

Human apoE exists in three different isoforms—apoE2, apoE3, apoE4—which differ in the amino acids at positions 112 and 158 [82]. In apoE2, both of these residues are cysteine, whereas in apoE4 both residues are arginine. In apoE3, which is the most common isoform, cysteine is at position 112 and arginine is at position 158. The apoE2 isoform is associated with Type III Hyperlipoproteinemia [83], whereas apoE4 is an important risk factor for Alzheimer’s disease [84].

ApoE contains two major structural domains linked by a hinge region. The N-terminal domain, spanning residues 1–191, contains four α-helices and forms a four-helix bundle structure [72,85]. Helix 4 is responsible for binding to receptors (residues 134–150 and arginine 172) and to HSPG (residues 142–147) [72,85]. It contains positively charged arginine and lysine residues, which interact with negatively charged binding sites in LDLR, LRP and HSPG. The C-terminal domain, spanning residues 225–299, contains amphipathic α-helices (residues 244–272) that bind to lipids and can also mediate cholesterol efflux by the ABCA1 transporter [72,78,85].

Only lipid-bound apoE can bind to LDL-related receptors with high affinity [86]. Lipid-bound apoE interacts with LDLR with approximately 20-fold greater affinity than apoB100 [73]. Receptor-binding regions in the N-terminal domain are shielded in its lipid-free form [85]. A two-step model has been proposed for apoE binding to lipoproteins. In a first step, the C-terminal domain binds lipids. In a second step, the N-terminal four-helix bundle undergoes a conformation change by opening up and exposing the LDLR- and HSPG-binding regions [72,85].

### 3.2. ApoE Mimetic Peptide Design

As apoE has several putative atheroprotective functions, many different types of apoE-based peptides have been reported [81]. One of the main goals in the design of these peptides is to facilitate the hepatic clearance of apoB-containing lipoproteins. As apoE can only bind to its receptor when bound to lipids, these peptides usually have not only the receptor-binding motif from the N-terminal domain of apoE, but also a lipid-binding region based on the C-terminal domain of apoE or some other sequence.

One of the earliest models of an apoE-mimetic peptide is Ac-hE18A-NH2 (Table 4) [87]. It contains 28 residues and was designed by linking the human apoE receptor-binding sequence LRKLRKRLLR (residues 141–150) (Figure 2D) with the 18A helix that avidly binds lipids (Figure 2A) [87]. The N- and C-terminus of the peptide were blocked by acetylation and amidation, respectively. This peptide bound to apoB-containing lipoproteins and increased their uptake into hepatocytes mainly via HSPG-mediated pathway and not by LDLR or related receptors [87,88]. Ac-hE18A-NH2 reduced hyperlipidemia in mouse [88,89,90] and rabbit models [91]. Furthermore, it stimulated endogenous apoE protein secretion from hepatocytes and macrophages [89,92]. When tested in apoE- and LDLR-null mouse models, Ac-hE18A-NH2 reduced atherosclerosis [89,93].

An analog of Ac-hE18A-NH2 peptide was designed by changing all the lysine residues in the receptor-binding region to arginine. This peptide called Ac-[R]hE18A-NH2 (Table 4) was even more potent in increasing lipoprotein uptake in cell culture studies [87]. Derivatives of Ac-[R]hE18A-NH2 containing α-aminohexanoic acid or other fatty acids (octanoic or myristic acid) attached to the N-terminus have also been described [96]. It was hypothesized that making these peptides more hydrophobic will enhance their ability to bind to atherogenic apoB-containing lipoproteins. The Myr-[R]hE18A-NH2 peptide (Table 4), containing myristic acid, was almost two-times more effective than Ac-hE18A-NH2 peptide in decreasing plasma cholesterol in apoE-null mice [96]. It also significantly reduced total and LDL-cholesterol (LDL-C) at lower doses than Ac-hE18A-NH2 in hypercholesterolemic cynomolgus macaques [96]. LDL-C was reduced by approximately 75% after 24 h and remained decreased for at least 3 days.

Handattu et al. developed a single-domain 18-residue apoE mimetic peptide called mR18L (Table 4) based on a cationic class L amphipathic helix [97,106]. In contrast to 18A, 18L helix has no negatively charged residues and the positively charged residues are all clustered at the center of the hydrophilic face. The mR18L peptide, which only has arginine and no lysine residues, promoted LDL uptake by hepatocytes [97] and had improved anti-inflammatory properties [106]. Furthermore, it decreased plasma cholesterol concentration and inhibited atherosclerosis in apoE-null mice [97]. The mR18L peptide also reduced plasma cholesterol concentration in LDLR-null mice as efficiently as Ac-hE18A-NH2 but was less effective in reducing atherosclerosis than Ac-hE18A-NH2 [89].

As amphipathic helices in the C-terminal domain of apoE have been shown to have potent cholesterol efflux activity [70,78], apoE mimetic peptides based on just this domain have also been developed. For example, the 26-residue helical peptide named ATI-5261 (Table 4), which contains amino acids corresponding to positions 238–266 of human apoE, was found to promote cholesterol efflux [98,99]. Ten residues of this peptide were changed compared to native sequence to make the peptide more helical and to increase its hydrophobic moment [99]. The ATI-5261 peptide stimulated macrophage cholesterol efflux in vitro [98,99] and macrophage RCT in apoE-null mice [99]. It also reduced atherosclerosis in apoE- and LDLR-null mice [99]; however, ATI-5261 had cytotoxic effects in muscles and it increased plasma triglycerides (TG) [101]. In a modified version of this peptide, named CS-6253 (Table 4), phenylalanine was changed to leucine on the hydrophobic face of the helix and positively charged arginine residues on the lipid-water interface were changed to neutrally charged citrulline residues [101]. CS-6253 was less cytotoxic and it stimulated macrophage cholesterol efflux via ABCA1 [100,101]. In addition to promoting cholesterol efflux through ABCA1, it also induced the release of cholesterol containing microparticles (50–350 nm) from cells and enhanced uptake by hepatocytes via SR-B1 [100,101].

Another apoE mimetic peptide, EpK (Table 4), with a N-terminal cysteine residue, contains the human apoE receptor-binding region (residues 141–150), followed by a six residues lysine linker and then the human apoE lipid-binding region (residues 234–254) [102]. It binds to HDL and promotes cholesterol efflux but does not reduce plasma cholesterol concentration in apoE-null mice [102]. EpK peptide, however, reduced atherosclerosis in apoE-null mice after lentivirus-mediated hepatic expression [107]. hEp (Table 4) is another peptide similar to EpK but contains a longer receptor-binding region (residues 131–162), which is directly linked to human apoE C-terminal lipid-binding region (residues 244–272) [103]. Lentivirus-mediated hepatic expression of hEp in apoE-null mice reduced plasma VLDL and LDL-C concentrations and also decreased atherosclerosis [103].

As previously reviewed, in addition to promoting apoB-containing lipoprotein uptake and cholesterol efflux, several apoE mimetic peptides, including Ac-hE18A-NH2, mR18L, EpK and hEp, have other atheroprotective properties, such as anti-inflammatory and anti-oxidative, not directly related to lipoprotein metabolism [81,108]. For example, the Ac-hE18A-NH2 peptide was shown to inhibit LPS-induced expression of vascular cell adhesion molecule-1 (VCAM-1), interleukin 6 (IL-6) and monocyte chemoattractant protein-1 (MCP-1) in human umbilical vein endothelial cells (HUVECs) and THP-1 macrophages [92]. It was also shown to reduce monocyte adhesion to HUVECs [92]. The Ac-hE18A-NH2 peptide also reduced plasma reactive oxygen species [89]. The AEM-2 (Ac-[R]hE18A-NH2 peptide with ɑ-aminohexanoic acid attached to the N-terminus) inhibited LPS-induced secretion of IL-6 and tumor necrosis factor-alpha (TNF-α) from THP-1 macrophages and also had anti-apoptotic effects in macrophages [109].

### 3.3. ApoE Mimetic Peptide Clinical Trials

Unlike apoA-I, full length apoE protein has not been investigated in human clinical trials, but two apoE mimetic peptides have been tested (Table 4). In early stage clinical trials, Ac-hE18A-NH2 (Table 4) (AEM28, Capstone Therapeutics) when infused IV in 51 patients in a phase 1b/2a study (dose range 1 mg/kg to 3.54 mg/kg) was shown to be safe [94,95] In a press release [95] was reported to rapidly decrease plasma TG and VLDL-cholesterol (VLDL-C) concentrations by approximately 50%. In primates, it was shown to reduce LDL-C up to 64% in 24 h following one intravenous dose of 5 mg/kg [110].

Due to the role of apoE in Alzheimer’s disease development and other neurologic diseases, apoE mimetic peptides have also been tested in various neurologic disease animal models and have yielded promising results [111]. One example of an apoE mimetic peptide designed for Alzheimer’s disease is CN-105 (Cerenova, LLC) (Table 4). It is short (5 residues) to facilitate its transfer across the blood–brain barrier and just contains three arginine residues that mimic the ligand-binding region of apoE. It was tested in 48 subjects in a phase 1 trial and shown to be safe [104]. Several other apoE-based peptides for neurologic diseases are actively being developed and have recently been reviewed [111].

## 4. ApoC-II

### 4.1. ApoC-II Biochemistry

ApoC-II plays a major role in TRL metabolism (Figure 1C), by acting as a cofactor of lipoprotein lipase (LPL), the main plasma enzyme that hydrolyses TG [10,112]. ApoC-II is mainly synthesized in the liver, but also in the intestine and in some other cell types like macrophages, but its role in these other sites is not clear [10]. After secretion into plasma, mature human apoC-II consists of 79 amino acids and has a molecular weight of 8916 Da. The plasma concentration of apoC-II is approximately 4 mg/dL (4.5 µM), but it can be significantly higher in patients with hypertriglyceridemia (HTG) [10].

Nuclear magnetic resonance structural studies of apoC-II/sodium dodecyl sulfate (SDS) micelle complexes showed that apoC-II contains three main domains [113,114]. The first is the N-terminal helix, which is a type A helix that spans residues 16–36 and is required for lipoprotein binding. The following 17 amino acids (residues 40–56) of apoC-II are mostly in a random coil configuration. The C-terminus (residues 63–76) forms a G-type helix [6] and is responsible for activating LPL. Although only the C-terminal part of apoC-II is required for LPL activation when using a synthetic lipid emulsion as the substrate [115], the lipid-binding regions in the N-terminus are also needed for complete LPL activation with its natural TRL substrate [116]. By site-directed mutagenesis, it has been shown that residues Tyr63, Ile66, Asp69 and Gln70 in the C-terminal helix are critical for LPL activation [117]. These residues are located on the same side of the helix and probably form a binding site for LPL [114,117]. Although the exact mechanism of LPL activation by apoC-II is unclear, it has been proposed that apoC-II helps to move the TG substrate molecules away from lipoproteins and into the active site of LPL [114]. In all-atom molecular dynamics simulation, apoC-II caused a thinning of the outer phospholipid monolayer and increased the interdigitation of TG with the acyl chains of phospholipids, thereby potentially making TG more available for LPL [118]. It was also recently proposed that apoC-II may regulate LPL activity in a surface pressure-dependent manner by keeping LPL tethered to the TRL surface during lipolysis [119].

### 4.2. ApoC-II Mimetic Peptide Design

The initial clinical rationale for the development of apoC-II mimetic peptides was for the treatment of Familial Chylomicronemia Syndrome (FCS) with severe HTG from apoC-II deficiency (OMIM 207750). Patients with FCS usually come to clinical attention due to an acute pancreatitis, which is a serious condition with a 5–6% mortality per episode [10]. In case of apoC-II deficiency, a favorable therapeutic response can often be seen after plasma exchange with fresh frozen plasma, which contains sufficient apoC-II protein to activate the patients endogenous LPL and acutely lower plasma TG concentrations [120]. Refining this concept and to make it possible to prophylactically treat such patients, apoC-II mimetic peptides (Table 5) were first developed [118,121], but as discussed below these peptides may also have broader applications.

The first apoC-II mimetic peptide, 18A-CII (Table 5) [121], contained instead of the long first helix of apoC-II, a shorter sequence based on the 18A peptide (Figure 2A) [21,122], which tightly binds to lipoproteins [21]. The second helix of 18A-CII was completely homologous to the C-terminal LPL activation domain of apoC-II [123]. This part of apoC-II by itself does not activate LPL very well, because of its inability to bind to lipoproteins and thus was linked to 18A by a proline residue [121]. Results from the isothermal titration calorimetry (ITC) studies in nearly undiluted human plasma, the natural matrix for LPL, showed that 18A-CII is a potent activator of LPL in both normolipidemic and HTG plasma samples [124,125]. Moreover, it was more potent than full-length apoC-II protein, which unlike the peptide inhibited lipolysis at higher concentrations [125]. Furthermore, 18A-CII along with exogenous LPL promoted the ex vivo lipolysis of TG in plasma from patients with apoC-II deficiency, and markedly lowered plasma TG concentrations in a mouse model of apoC-II deficiency (Table 5) [126]. In mice, 18A-CII was found to increase the uptake of fatty acids from TG lipolysis into peripheral tissues, such as skeletal muscle and adipose tissue, but it reduced hepatic uptake of TG [127].

Recently, a second generation of apoC-II mimetic peptide called D6PV has been described [118] (Table 5). As apoC-II mimetic peptide treatment for FCS would likely be a chronic therapy, there was a concern that the use of the artificial 18A helix (Figure 2A) could eventually lead to the production of blocking antibodies that would interfere with LPL activation [122]. Instead of 18A, the first helix of D6PV (Figure 2E) is based on the native random coil region of apoC-II, which is next to the LPL activation domain (Figure 2F). Several amino acids substitutions were made in the random coil region to enhance the ability of this bi-helical peptide to bind to lipoproteins (Figure 2E) [118].

Ex vivo assays showed that D6PV had similar potency as 18A-CII in activating LPL and it markedly lowered plasma TG levels by as much as 90% after 1 h in apoC-II-deficient mice [118]. Unexpectedly, D6PV also significantly lowered TG in h*APOC3*-transgenic (Tg) mice, which have normal levels of apoC-II. In vitro studies demonstrated that D6PV displaces apoC-III from human VLDL and relieves inhibition of LPL by apoC-III. Additionally, h*APOC3*-Tg mice treated with D6PV had increased renal clearance of apoC-III, resulting in a 80% reduction in apoC-III and a 65% reduction in apoB in plasma. When whole-body inducible *Lpl* knockout (i*Lpl^−/−^*) mice were treated with D6PV, TG levels decreased by about half, which confirms that the TG-lowering effect of the peptide can occur without LPL activation by the lowering of apoC-III concentration, which besides inhibiting LPL is also known to block hepatic uptake of lipoproteins [128].

When tested in non-human primates, D6PV had good subcutaneous bioavailability and a relatively long terminal half-life (42–50 h), due to binding to HDL, which serves as a long-term reservoir of the peptide. Based on these findings, D6PV appears to act as both a mimetic for apoC-II and an antagonist for apoC-III. It may, therefore, be useful for other forms of HTG besides apoC-II deficiency and also for other CV diseases given the favorable results of clinical trials for Volanesorsen, an anti-sense oligonucleotide drug that blocks apoC-III production [129,130].

### 4.3. ApoC-II Mimetic Peptide Clinical Trials

Research on apoC-II mimetic peptides (Table 5) is relatively new compared to the other apolipoprotein mimetics, and no clinical trials of apoC-II mimetic peptides have been reported [129]. D6PV was developed under a collaboration between NIH investigators and Corvidia Therapeutics Inc. [118,131], which was recently acquired by NovoNordisk. Ongoing clinical trials on anti-sense oligonucleotides against apoC-III, may prove to be informative on whether the lowering of plasma apoC-III levels, in general, is useful for CV risk reduction [129]. In addition, clinical trials on other TG-lowering therapies may also help inform on the overall value of TG lowering for CV diseases risk reduction [129].

## 5. Discussion and Conclusions

As we reviewed, much has been learned about lipoprotein metabolism from research related to apolipoprotein mimetic peptides and several of these peptides have moved into early stage clinical trials. Ultimately, success into developing any of these peptides into an approved therapy will mainly depend on two factors, namely the validity of the target for the peptides and the pharmacokinetic and pharmacodynamic factors related to the delivery of the peptide.

In terms of the validity of the target for apoA-I mimetic peptides, there is still great uncertainty about whether drugs that modulate HDL can effectively alter the development or regression of atherosclerosis [132]. To date all the investigational drugs, such as CETP-inhibitors, have failed to decrease CV events based on their HDL-C raising effect [133]. If CSL-112, the intact apoA-I protein purified from human plasma and complexed with phospholipids, succeeds in its now ongoing phase 3 clinical trials, it will likely reinvigorate efforts related to apoA-I mimetic peptides. Short synthetic peptides would essentially have no risk for viral disease transmission unlike apoA-I purified from plasma, be less costly to produce, and some would be more potent in mediating cholesterol efflux than the full-length apoA-I protein [58].

As for apoE mimetic peptides, their main target is for lowering LDL-C, a well-established therapeutic strategy that has proven to be successful for many other types of approved drugs like statins and proprotein convertase subtilisin/kexin type 9 (PCSK9)-inhibitors [96,110]. However, even if these peptides efficiently lower plasma LDL-C, there is still some uncertainty about the fate of the LDL particles lowered by these peptides. If the bulk of the LDL is delivered to the liver, it will likely be beneficial, but if the peptide also leads to their increased deposition into the vessel wall, it could even accelerate the development of atherosclerosis. It will also be challenging to develop apoE mimetic peptides as new therapy given how successfully one can already lower LDL-C with statins and PCSK9-inhibitors.

There is growing evidence that TG, the target for the apoC-II mimetic peptides, is involved in the process of atherosclerosis [129], but there is still some uncertainty about this. For example, eicosapentaenoic acid ethyl ester in the REDUCE-IT trial did effectively lower TG levels and CV events when used on top of statins [134], but its beneficial effect on CV diseases risk reduction appears to be independent of its TG-lowering effect. Lowering TG for prevention and/or treatment of acute pancreatitis by apoC-II mimetics will likely prove to be beneficial, but this is a much smaller population at risk [10,112].

Peptides also have many challenges in terms of drug development, because of their pharmacodynamic and pharmacokinetic features [135]. In general, peptides are costly to produce and require difficult routes of delivery, such as intravenous or subcutaneous. They also frequently have a very short half-life due to their small size and rapid renal elimination. Nevertheless, there are over 60 different peptides that are now approved for human use for a wide variety of conditions [136]. There have also been great advances in peptide chemistry, and it is now much easier and less costly to produce synthetic peptides. Apolipoprotein mimetic peptides have the advantage over many other therapeutic peptides in that they typically have a relatively long half-life, because they reside on lipoproteins, which in the case of HDL and LDL circulate in human plasma for several days, e.g., apoC-II mimetic peptides [112,118]. Advances in peptide design and delivery have also led to the development of several peptides drugs that can be taken orally [137]. Thus, the ongoing progress in peptide chemistry and formulation may make it feasible to develop apolipoprotein mimetic peptides into an approved therapy.

In summary, many decades of work on apolipoproteins have led to the development of apolipoprotein mimetic peptides. To date, such work has aided in our understanding of the biological role of apolipoproteins and with more work may eventually lead to new therapies for CV and other diseases. Ongoing work on other apolipoprotein mimetic peptides besides those described here may reveal new structural motifs to mimic the other functions of apolipoproteins that could also have therapeutic value.

## Figures and Tables

**Table 1:** Characteristics of apoA-I, apoE and apoC-II.

Apo	MW (kDA)	Number of residues	Plasma Concentration (mg/dL)	Site of Synthesis	Main Functions	Source
ApoA-I	28.3	243	~120–140	Liver, intestine	Structural protein for HDL, activates LCAT	[2]
ApoE	34.0	299	~4–7	Liver, intestine, macrophages, brain (astrocytes), skin	Ligand for LDLR, LRP and HSPG	[9]
ApoC-II	8.9	79	~4	Liver, intestine, macrophages	Co-factor for LPL	[10]

**Table 2:** ApoA-I mimetic peptides.

Agent	Number of residues	Sequence	Main Features	Route	Main Findings	Stage	Source
18A	18	D-W-L-K-A-F-Y-D-K-V-A-E-K-L-K-E-A-F	Forms α-helix	IV	Good lipoprotein binding	Pre-clinical	[21]
D-4F	18	Ac-D-W-F-K-A-F-Y-D-K-V-A-E-K-F-K-E-A-F-NH2	4F made with D-amino acids	PO	Increased hydrophobicity over 18A	Phase 2	[22]
L-4F	18	Ac-D-W-F-K-A-F-Y-D-K-V-A-E-K-F-K-E-A-F-NH2	4F made with L-amino acids	IV, SC	Similar to D-4F but susceptible to proteolysis	Phase 2	[23]
ETC-642	22	P-V-L-D-L-F-R-E-L-L-N-E-L-L-E-A-L-K-Q-K-L-K	Single helix complexed with DPPC	IV	Activates LCAT	Phase 1	[24]
5A	37	D-W-L-K-A-F-Y-D-K-V-A-E-K-L-K-E-A-F-P-D-W-A-K-A-A-Y-D-K-A-A-E-K-A-K-E-A-A	Ala substitutions in second helix	IV	ABCA1 specific and less cytotoxic	Phase 1	[17,25,26]

**Table 3:** Other HDL related therapies.

Agent	Formulation	Route	Findings	Stage	Source
ETC-216	Recombinant apoA-I with ARG173CYS substitution reconstituted with phospholipids	IV	No plaque reduction	Phase 2	[54]
CSL112	ApoA-I purified from plasma and reconstituted with phospholipids	IV	Ongoing (AEGIS-II)	Phase 3	[57]
CER-001	Recombinant apoA-I reconstituted with sphingomyelin and DPPC	IV	No plaque reduction	Phase 2	[62]

**Table 4:** ApoE mimetic peptides.

Agent	Number of residues	Sequence	Main Features	Route	Main Findings	Stage	Source
Ac-hE18A-NH2/AEM28/AEM-28–08	28	Ac-L-R-K-L-R-K-R-L-L-R-D-W-L-K-A-F-Y-D-K-V-A-E-K-L-K-E-A-F-NH2	Receptor-binding region of apoE linked to 18A	IV	Increases hepatic removal of apoB-containing lipoproteins	Phase 1a and 1b/2a	[94,95]
Ac-[R]hE18A-NH2	28	Ac-L-R-R-L-R-R-R-L-L-R-D-W-L-K-A-F-Y-D-K-V-A-E-K-L-K-E-A-F-NH2	Lys changed to Arg in receptor-binding region of apoE	IV	Improves lipoprotein uptake over Ac-hE18A-NH2 in cell culture studies	Pre- clinical	[87]
Myr-[R]hE18A-NH2	28	Myristyl-L-R-R-L-R-R-R-L-L-R-D-W-L-K-A-F-Y-D-K-V-A-E-K-L-K-E-A-F-NH2	Myristic acid added to Ac-[R]hE18A-NH2	IV	More effective than Ac-hE18A-NH2 in reducing LDL-C	Pre- clinical	[96]
mR18L	18	Ac-G-F-R-R-F-L-G-S-W-A-R-I-Y-R-A-F-V-G-NH2	Cationic class L amphipathic α-helix	IV, IP, PO	Single domain peptide that reduces plasma cholesterol after oral administration	Pre- clinical	[89,97]
ATI-5261	26	Ac-E-V-R-S-K-L-E-E-W-F-A-A-F-R-E-F-A-E-E-F-L-A-R-L-K-S-NH2	Amphipathic helical peptide based on C-terminal lipid-binding region of apoE	IP	Promotes cholesterol efflux	Pre- clinical	[98,99]
CS-6253	26	Ac-E-V-Cit-S-K-L-E-E-W-L-A-A-L-Cit-E-L-A-E-E-L-L-A-Cit-L-K-S-NH2	Phe and Arg changed to Leu and Cit, respectively, compared to ATI-5261	IV	Promotes cholesterol efflux and is less cytotoxic than ATI-5261	Pre- clinical	[100,101]
EpK	38	C-R-R-K-L-R-K-R-L-L-R-K-K-K-K-K-K-Q-V-A-E-V-R-A-K-L-E-E-Q-A-Q-Q-I-R-L-Q-A-E	Receptor-binding region of apoE connected via Lys linker to apoE lipid-binding region	_	Recombinantly produced peptide that promotes cholesterol efflux but does not reduce plasma cholesterol	Pre- clinical	[102]
hEp	61	E-E-L-R-V-R-L-A-S-H-L-R-K-L-R-K-R-L-L-R-D-A-D-D-L-Q-K-R-L-A-V-Y-E-E-Q-A-Q-Q-I-R-L-Q-A-E-A-F-Q-A-R-L-K-S-W-F-E-P-L-V-E-D-M	Modified EpK with longer receptor-binding region and lipid-binding region of apoE	_	Reduces plasma VLDL and LDL-C	Pre- clinical	[103]
CN-105	5	Ac-V-S-R-R-R-NH2	Derived from the polar face of the receptor-binding region of apoE	IV	Reduces neuro-inflammation and improves survival and functional outcomes of ischemic stroke, traumatic brain injury, and intracranial hemorrhage in mice	Phase 2	[104,105]

**Table 5:** ApoC-II mimetic peptides.

Agent	Number of Residues	Sequence	Main Features	Route	Main Findings	Stage	Source
18A-CII	40	D-W-L-K-A-F-Y-D-K-V-A-E-K-L-K-E-A-F-P-A-M-S-T-Y-T-G-I-F-T-D-Q-V-L-S-V-L-K-G-E-E	18A linked to LPL-activation domain of apoC-II	IV, IP, SC	Activates LPL	Pre-clinical	[121]
D6PV	40	D-Y-L-K-E-V-F-E-K-L-R-D-L-Y-E-K-F-T-P-A-V-S-T-Y-T-G-I-F-T-D-Q-V-L-S-V-L-K-G-E-E	Both first and second helices are based on apoC-II sequence	IV, IP, SC	Activates LPL and lowers apoC-III	Pre-clinical	[118]
